# Supplementary material for: Associations of Caregiver Cooking Skills with Child Dietary Behaviors and Weight Status: Results from the A-CHILD Study
Source: Nutrients. 2021 Dec 18;13(12):4549. doi: 10.3390/nu13124549 (PMC8704868; doi:10.3390/nu13124549)
Supplement: Supplementary file 1 [file nutrients-13-04549-s001.zip › nutrients-1410722-SI.pdf]

Supplementary Table S1. Spearman correlation coefficients for items on the cooking skills scale

		1	2	3	4	5
All (*n* = 5257)						
1	Able to peel fruits and vegetables	1.00				
2	Able to make stir-fried meat and vegetables	**0.77**	1.00			
3	Able to make miso soup	**0.72**	**0.84**	1.00		
4	Able to make stewed dishes	**0.60**	**0.71**	**0.72**	1.00	
5	Like to cook	**0.20**	**0.22**	**0.20**	**0.26**	1.00
Mother (*n* = 4768)						
1	Able to peel fruits and vegetables	1.00				
2	Able to make stir-fried meat and vegetables	**0.76**	1.00			
3	Able to make miso soup	**0.70**	**0.83**	1.00		
4	Able to make stewed dishes	**0.57**	**0.69**	**0.70**	1.00	
5	Like to cook	**0.18**	**0.19**	**0.17**	**0.24**	1.00
Father (*n* = 414)						
1	Able to peel fruits and vegetables	1.00				
2	Able to make stir-fried meat and vegetables	**0.78**	1.00			
3	Able to make miso soup	**0.74**	**0.87**	1.00		
4	Able to make stewed dishes	**0.61**	**0.72**	**0.77**	1.00	
5	Like to cook	**0.47**	**0.50**	**0.49**	**0.51**	1.00

Boldface indicates statistical significance ($p<0.05$).
